# Comprehensive assessment of serum estradiol impact on selected physiologic markers observed during in-vitro fertilization and embryo transfer cycles

**Published:** 2009-10-20

**Authors:** Grace Wing Shan Kong, Lai Ping Cheung, Christopher John Haines, Po Mui Lam

**Affiliations:** 1 Department of Obstetrics and Gynecology, Prince of Wales Hospital, Shatin, Hong Kong SAR; 2 Department of Obstetrics and Gynecology, The Chinese University of Hong Kong, Hong Kong SAR

**Keywords:** Estradiol, in-vitro fertilization, embryo transfer

## Abstract

**Objective:**

This investigation assessed the effect of serum estradiol levels on outcomes of in-vitro fertilization and embryo transfer (IVF) cycles.

**Materials and method:**

This was a retrospective cohort study of 1123 IVF cycles comparing impact of estradiol (E_2_) levels on follicular development, fertilization, embryo quality, implantation, pregnancy rate, miscarriage rate, and selected obstetric complications.

**Results:**

We found high serum E_2_ levels to be significantly associated with increased number of mature follicles and mature oocytes retrieved (*p*<0.01, for both). E_2_ levels were also associated with more viable and good-quality embryos (*p*<0.01). There was no significant impact of E_2_ on oocyte maturation, fertilization rate, embryo quality, or overall pregnancy rates. Moreover, high E_2_ levels were significantly associated with higher implantation rates and reduced incidence of miscarriage (*p*<0.05, for both).

**Conclusion:**

Within the safety range in clinical practice, our data demonstrate a generally positive effect of high serum E_2_ on selected IVF parameters.

## Introduction

In the clinical practice of in-vitro fertilization and embryo transfer (IVF), controlled ovarian hyperstimulation (COH) is a common technique to maximize the number of mature oocytes retrieved. This approach must be balanced with the recognized risks of ovarian hyperstimulation syndrome (OHSS), which can result if follicular recruitment is too robust. Together with the multifollicular development, COH is inevitably associated with a supra-physiological serum level of E_2_ which in turn may affect endometrial receptivity [1]. The elevated serum E_2_ estradiol levels during COH may therefore be associated either with an increased chance of pregnancy (reflecting a better ovarian response), or an impaired reproductive outcome secondary to altered endometrial receptivity.

Possible associations between serum E_2_ levels and IVF outcomes have been the focus of research interest for many years. Several studies have illustrated the detrimental effect of high serum E_2_ levels on IVF outcome [2, 3] although this finding has not been confirmed in others [4–8]. Indeed, some investigators have noted that serum E_2_ can positively predict IVF outcomes via the retrieval of a greater number of oocytes [9–11]. Conflicting results of previous publications might be related to differences in outcome measures as well as threshold values used to define “high” E_2_ levels. While most investigators have focused on the implantation and pregnancy rates which are only the final steps, an IVF cycle indeed involves a sequence of highly synchronized events each of which must proceed optimally. Not only endometrial receptivity and embryo implantation, but also follicular development, fertilization, and embryo quality are all important keys for determining success of an IVF cycle.

This study aims to present a comprehensive assessment of the impact of serum E_2_ on the different components of an IVF cycle. We compare the impact of serum estradiol levels measured on the day of ovulatory dose of human chorionic gonadotrophin (hCG) on follicular development, fertilization, embryo quality, implantation, pregnancy rate, miscarriage rate, and complications such as OHSS.

## Methods

This retrospective cohort study encompassed 1123 IVF cycles performed during January 2005 to December 2007 at the Assisted Reproductive Unit (Hong Kong). This is the tertiary referral centre affiliated with the Department of Obstetrics and Gynecology, The Chinese University of Hong Kong. Since there was no human subjects tested or interviewed specifically for the purpose of this investigation, it was determined that IRB approval was not required for our retrospective study. All IVF cycles performed during the above period were analyzed; no cases were excluded. The causes of infertility for women undergoing IVF cycles included tubal, male, endometriosis, unexplained and mixed factors. Intracytoplasmic sperm injection (ICSI) was carried out in couples with severe semen abnormalities.

### Controlled Ovarian Hyperstimulation (COH) Protocol

All subjects received a standard protocol for ovulation induction. Pituitary suppression was achieved via long (luteal) gonadotropin releasing hormone agonist (GnRHa) down-regulation protocol. Buserelin nasal spray (Suprecur, Hoechst, Germany) 600 mcg daily was administered for at least 14d from the mid-luteal phase of the preceding cycle. Complete pituitary desensitization was confirmed by low serum luteinizing hormone (LH < 10 IU/L) and estradiol (E_2_ < 200 pmol/L) concentrations. Patients also had an ultrasound examination to exclude functional ovarian cysts and verify that endometrial thickness was <5mm. Once adequate downregulation was confirmed, ovarian stimulation commenced with human menopausal gonadotropins (hMG) (Pergonal, Serono, Aubonne/Switzerland) or recombinant follicle stimulating hormone (rFSH) (Gonad-F, Serono, Aubonne/Switzerland; or Puregon, Organon, Oss, Holland). For patients undergoing their first IVF cycles, initial doses of hMG (or equivalent dose of rFSH) were 225, 300, or 375IU/d for female age groups of < 33yrs, 33–35yrs, and >35yrs, respectively. The starting dose was reduced to 150IU/d for anticipated high responders (i.e., PCO patients). Alternatively, baseline gonadotropin dose was increased to 450IU/d for anticipated poor responders, such as those with elevated basal serum FSH levels. For patients undergoing subsequent IVF cycles, the starting doses were influenced by ovarian response in the prior cycle/s as well as patient age. Neither antral follicle count nor ovarian volume was considered in dose determination.

Ovarian response was monitored by transvaginal ultrasound and serum E_2_ concentrations from stimulation day 6 onwards. The dose of gonadotropins was adjusted during the stimulation depending on the ovarian response. When follicular recruitment was considered adequate (defined by the presence of at least three mature follicles 18mm in diameter), an ovulatory dose of hCG (Profasi, Serono, Aubonne/Switzerland) 5,000 IU was given and transvaginal oocyte retrieval was performed approximately 36h later. All patients received a titrated dose of diazepam and pethidine for analgesia. For patients with poor ovarian response (defined as less than three mature follicles on ultrasound), the cycle was cancelled and retrieval was not attempted. Embryo transfer was performed three days after oocyte retrieval and surplus embryos were cryopreserved. Patients undergoing embryo transfer received intramuscular hCG or vaginal progesterone for luteal phase support beginning on the day of oocyte retrieval. For those with excessive ovarian response (defined as >20 oocytes retrieved, or 15–20 oocytes retrieved but with risk factors for OHSS such as known PCO or past history of OHSS), all viable embryos were cryopreserved instead so as to minimize the risk of OHSS.

### Outcome Measures

IVF cycle outcome was assessed by urine pregnancy test performed 10d after the final dose of hCG (luteal phase support) or two weeks after embryo transfer, whichever occurred later. If a positive test was reported, vaginal ultrasound of the pelvis would be performed two weeks later to assess the site, the number, and the viability of gestation. Pregnancy rate was defined as positive urine pregnancy test per embryo transfer, while implantation rate was defined as number of gestational sacs per embryo transferred. The pregnancy outcomes, such as miscarriage and live birth were also recorded, along with multiple gestation rate and complications.

Clotted blood samples (5ml) were collected by peripheral venipuncture at the conclusion of follicular recruitment (on the day of ovulatory dose of hCG) for serum E_2_ measurements. Serum estradiol levels were quantified by using a competitive immunoassay with direct chemiluminesent technology (Bayer, Tarrytown, NY, USA). Interassay coefficients of variation of the assay were 9.8%, 4.2%, and 8.7% at concentrations of 250, 859, and 2830 pmol/L respectively.

Data on patient characteristics, ovarian stimulation protocol and embryology were also collected. These included ages of patients, type, duration, and cause of infertility, ovarian reserve assessment (CD#3 serum FSH levels), number of previous IVF cycles, duration and total dose of gonadotropin treatment, endometrial thickness on day of ovulatory hCG, number of ovarian follicles >15 mm in diameter, number of mature oocytes and total oocytes retrieved, fertilization rate, and number of viable embryos and good-quality embryos (which were defined as four-cell or more cleaved embryos, seven-cell or more cleaved embryos and blastocysts transferred 48h, 72h and 120h after retrieval, respectively). The number of embryos transferred per patient was also reviewed.

### Statistics

Statistical analysis was carried out using SPSS (version 16). Continuous data were expressed as mean ± standard deviation (SD). One-way analysis of variance (ANOVA) with Bonferroni correction for multiple comparisons was used to analyze continuous data and chi-square test was used to analyze categorical data, where appropriate. P<0.05 was considered statistically significant.

## Results

A total of 1123 IVF cycles were studied. Among them, 418 (37.2%) cycles had ICSI performed for severe semen abnormalities; 607 (54.1%) cycles were the first IVF trial for the women. The women aged 35.8 ± 3.7 years and the baseline serum FSH level was 7.7 ± 2.7 mmol/L. Concerning the outcomes, the mean (±SD) number of total oocytes and mature oocytes retrieved per cycle were 9.8 ± 5.6 and 7.8 ± 4.6, respectively. Mean (±SD) number of viable embryos and good embryos per cycle were 2.7 ± 2.2 and 2.0 ± 2.1, respectively. The overall implantation rate was 22.6% while the overall pregnancy rates per cycle initiated, per cycle with oocyte retrieval, and per cycle with embryo transfer were 31%, 31% and 36%, respectively.

The mean, 25 percentile, median and 75 percentile of serum E_2_ levels on day of hCG administration were 13,200, 6000, 10500 and 17300 pmol/l, respectively. Based on these percentiles, the IVF cycles were categorized into four groups: Group 1 (243 cycles with E_2_ < 6000 pmol/L), Group 2 (393 cycles with E_2_ = 6000–12000 pmol/L), Group 3 (231 cycles with E_2_ = 12000–18000 pmol/L), and Group 4 (256 cycles with E_2_ > 18000 pmol/L). In Group 4 (the high responder group), 54 cycles (20%) underwent elective cryopreservation of all embryos in view of anticipated high risk of OHSS. Among them, 18 (33%) cycles did develop OHSS. The incidence of OHSS for the whole cohort was 12.5% (140 cycles), and 1.2% (13 cycles) were severe cases.

Women in Group 4 were younger (34.7 ± 3.5 vs. 37.0 ± 3.8 & 35.8 ± 3.6yrs, *p*<0.01), had lower average baseline FSH level (6.7 ± 1.6 vs. 8.7 ± 2.9 & 7.9 ± 2.6 IU/L, *p*<0.01) and consumed a lower gonadotropin dose during IVF (3021.4 vs. 4352.9 & 3824.4 IU, *p*<0.01) compared to patients in Group 1 and 2, respectively. However, there were no significant differences among the groups in other potential covariates, including frequency of PCO diagnosis, number of previous IVF cycles, fertilization method, and cancellation rate due to poor ovarian response (see Table 1).

Follicular development in Group 4 demonstrated an increased number of follicles >15mm in diameter (12.4 ± 3.7 vs. 4.4 ± 1.9, 6.9 ± 2.6 & 9.3 ± 3.2, *p*< 0.01), more oocytes retrieved (15.3 ± 6.1 vs. 5.3 ± 3.0, 8.3 ± 3.6 & 10.9 ± 4.5, *p*< 0.01), and a higher average number of mature oocytes retrieved (12.2 ± 5.1 vs. 4.3 ± 2.6, 6.6 ± 3.0 & 8.7 ± 3.8, *p*< 0.01) compared to s 1, 2 and 3, respectively. However, there were no significant differences among study groups regarding oocyte maturation rate or average number of cycles where poor oocyte quality was observed (see Table 2).

The fertilization rate was 60%, which was not significantly different among these 4 groups with different E_2_ levels. However, IVF cycles in Group 4 had a higher mean number of viable embryos (3.9 ± 3.1 vs. 1.7 ± 1.5 & 2.5 ± 1.7, *p*<0.01), a higher average number of good embryos (2.9 ± 2.8 vs. 1.2 ± 1.4 & 1.8 ± 1.7, *p*<0.01), but a reduced proportion of viable embryos per fertilized oocyte (0.49 ± 0.27 vs. 0.58 ± 0.36 & 0.58 ± 0.30, p< 0.01) compared to Group 1 and 2, respectively. However, there were no significant differences in proportion of good embryos per fertilized oocyte among the study groups (see Table 2).

There were no significant differences in pregnancy rates, live birth rates per cycle, or miscarriage and multiple gestation rates per pregnancy among the four groups. However, IVF cycles in Group 4 demonstrated a higher implantation rate (0.28 ± 0.38 vs. 0.20 ± 0.33, *p*=0.04) compared to Group 2. Also, Group 4 was associated with lower miscarriage rate (12.2 vs. 23.6% & 24.2 %, *p*<0.05) compared to Groups 1 and 2, respectively. As expected, the complication rate of OHSS was significantly higher in Group 4 (22 vs. 4.5% & 9.7%, *p*< 0.01) compared to Groups 1 and 2, respectively (see Table 3).

For the sub-set of patients with extremely high E_2_ levels accompanied by elective embryo cryopreservation (no fresh transfer), these cycles were associated with more follicles, more total oocytes retrieved, more mature oocytes retrieved and more viable embryos. However, oocyte maturation, number of cycles with poor oocyte quality, fertilization rate, proportion of viable embryos per fertilized oocyte, number of good quality embryos, and proportion of good embryos per fertilized oocyte were not significantly different from the other 4 groups.

A subgroup analysis was also performed for patients with a favorable prognosis, (i.e., those age <38yrs and with CD#3 FSH level <10mmol/L). A total of 664 IVF cycles met this criteria and were eligible for analysis; these were stratified into four categories according to the same E_2_ threshold levels as used in the overall analysis. Tables 4 and Table 5 summarize IVF outcomes in this subgroup of favorable prognosis patients. The results were similar to the overall group, but with significant improvement in follicular development and number of embryos. There was also a reduced rate of miscarriage (but an increased risk of OHSS) in Group 4 compared to Groups 1 and 2. However, there were no significant differences among groups in fertilization rate, implantation rate, pregnancy and live birth rates per cycle, or miscarriage and multiple gestation rates.

## Discussion

It is well established that E_2_ is essential in an IVF cycle by protecting developing follicles from atresia and stimulating endometrial growth for subsequent implantation and further pregnancy. However, the associations between various serum E_2_ levels and particular IVF outcomes have remained controversial. Low serum estradiol level is apparently associated with poor IVF outcomes. However, the possible adverse impact of elevated serum E_2_ level on the IVF outcomes is still under debate. A meta-analysis published in 2004 regarding the associations between serum estradiol level on day of hCG administration and pregnancy after IVF drew no conclusions [12]. The current available evidence, though reasonably extensive, does bring some limitations with the difference in the cut-off values for E_2_ level being the obvious concern.

Earlier work has shown that high serum E_2_ levels in the setting of IVF might indicate increased ovarian response [9–11], increased risk of OHSS [11] and/or reduced endometrial receptivity [2, 3]. Indeed, serum E_2_ level measured on day of ovulatory dose of hCG is a common clinical indicator for elective cryopreservation of all viable embryos as a prophylactic measure to reduce risk of OHSS, although different assisted reproductive centres have developed different E_2_ threshold levels for this. Studies with lower cut-off for serum E_2_ level above which all embryos were cryopreserved could demonstrate the impact of E_2_ level on ovarian response only, but not its impact on OHSS development or endometrial receptivity. However, it would be unethical to place patients at risk of developing severe OHSS by allowing fresh embryo transfer in cycles with an extremely high serum E_2_ level to reveal the impact on endometrial receptivity. The incidence of severe OHSS in our unit during the study period was 1.2%. Within this safety range in clinical practice, our retrospective study has evaluated the impact of serum E_2_ levels on day of hCG administration on the outcomes from IVF in a comprehensive manner.

The relatively higher serum E_2_ levels measured from controlled ovarian hyperstimulation reflected a better ovarian response of IVF, and as expected, these cycles were from patients with better ovarian reserve as suggested by their younger ages and lower baseline FSH levels. Most importantly, our study has observed a negative relationship between the consumption of gonadotropin and serum E_2_ levels at the time of hCG administration. This finding suggests that a high E_2_ level is not necessarily a result of high-dose gonadotrophin stimulation. Indeed, for women with good ovarian reserve, the lower dose of gonadotrophin is enough to trigger an adequate ovarian response with high serum E_2_ levels. Therefore, the dosage of gonadotrophin used in controlled ovarian hyperstimulation should be adjusted, especially for women with good prognostic factors so as to avoid further increase in serum estradiol level which may result in potential detrimental effect on the IVF cycle and OHSS.

Although the incidence of severe OHSS (1.2%) in our unit during the study period was acceptable, the incidence of overall OHSS was too high. After this retrospective analysis, we reevaluated our practice and implemented some changes to reduce the risk of OHSS. Specifically, we have modified the starting doses of gonadotropin to be calibrated not only by patient age and previous treatment response, but also by antral follicle count. This has resulted in starting doses of gonadotropins now being lower for most paitents. Coasting in selected cycles with high risk of OHSS will also be considered, all IVF patients will have vaginal progesterone (rather than intramuscular hCG) for luteal phase support.

In agreement with previous studies which showed the relationship between high serum E_2_ level and good ovarian response [9–11], our data demonstrate the improvement of follicular development as well as the quality and quantity of oocyte retrieval in term of number and maturity of oocytes retrieved. We specifically investigated the relationship between the proportion of mature oocyte per oocyte retrieved and the serum E_2_ levels, and found that there were no significant associations between them. This observation suggests that the higher E_2_ levels only result in more oocytes (and so an increased number of mature oocytes proportionally), but does not promote the maturation of oocytes *per se*. Concerning 2*pn* fertilization, our study did not observe any significant differences in fertilization rates among the four groups with different levels of serum E_2_. Thus, our findings showed high serum E_2_ were associated with a better ovarian response but had no effect on fertilization rate itself.

Although some studies have commented on the increased embryo yield in patients with high serum E_2_ levels [3, 9], ours is the first description of the impact of serum E_2_ levels on embryo quality. In this investigation, we found that high serum E_2_ levels were associated with increased number of viable embryos and good-quality embryos per cycle. However, the proportion of viable embryos per fertilized embryo was indeed lower in the group with high E_2_ levels. These observations suggest that high E_2_ levels are associated with more oocytes retrieved and so more viable or good-quality embryos proportionally, but they are not associated with improvement in the quality of embryo. Therefore, we do not anticipate any impact of these high serum E_2_ levels on the outcome of the subsequent cryopreserved embryo cycles. However, this question remains to be answered in further analysis.

The impact of high E_2_ level (measured on day of hCG administration) on implantation is controversial. Some authors have reported that high E_2_ level could be associated with no change or even an increased implantation rate [9, 11, 13, 14], while others reported a detrimental effect and impaired implantation [1, 2, 15, 16]. Arslan *et al* analyzed the cumulative E_2_ effect (using the “area under the curve” approach) which maybe a better reflection of the total E_2_ exposure of both oocytes and endometrium [17]. In parallel with our work, they found that different levels of E_2_ exposure did not likely affect oocyte and embryo quality [17]. However, they demonstrated detrimental effects of high cumulative E_2_ exposure on embryo implantation [17]. Postulated mechanisms for reduced implantation include impaired endometrial receptivity [17, 18], altered E_2_ to progesterone ratio [19], and reduction in the nuclear receptor for E_2_ and progesterone in endometrial stroma and glands [20]. Morphological and biochemical changes in secretory endometrium have also been demonstrated [21–24]. Within the safety range for OHSS in our clinical practice, we indeed demonstrated a positive effect of high serum E_2_ levels on IVF outcome in terms of a higher implantation rate and a lower miscarriage rate, which is a reflection of endometrial receptivity.

In conclusion, we have found that a high serum E_2_ level at the time of hCG administration is a marker of a robust ovarian response to controlled ovarian hyperstimulation and is accompanied by more mature follicles, more mature oocytes retrieved, and a higher yield of viable and good-quality embryos. There is no significant impact of serum E_2_ level on fertilization rate, maturation of oocytes and quality of embryo per se, or the overall pregnancy rates. The high E_2_ level did not adversely affect the endometrial receptivity but indeed is associated with a higher implantation rate and a lower miscarriage rate.

## Figures and Tables

**Table 1:** Patient characteristics observed among four groups of in-vitro fertilization cycles as a function of serum estradiol.

Characteristics	Group 1 (n=243)	Group 2 (n=393)	Group 3 (n=231)	Group 4 (n=256)
Age (yrs)	37.0 ± 3.8#	35.8 ± 3.6#	35.5 ± 3.5	34.7 ± 3.5
Day 3 FSH (IU/L)	8.7 ± 2.9#	7.9 ± 2.6#	7.1 ± 1.9	6.7 ± 1.6
PCO	4 (1.6%)	22 (5.6%)	11 (4.8%)	16 (6.3%)
First trial of IVF	122 (50.2%)	211 (53.7%)	115 (49.8%)	159 (62.1%)
Fertilization with ICSI	89 (36.6%)	152 (38.7%)	84 (36.4%)	93 (37.2%)
Total dose of gonadotropin (IU)	4353#	3824#	3512*	3021
Cancellation of cycle	3 (1.2%)	1 (0.3%)	1 (0.4%)	1 (0.4%)

Values are numbers (percentages) of cycles or mean ± standard deviation.

n = number of cycles of in-vitro fertilization

FSH: Follicle stimulation hormone

PCOS: Polycystic ovary syndrome

IVF: In vitro fertilization

ICSI: Intracytoplasmic sperm injection

* (*p*<0.05) and

^#^ (*p*<0.01) indicate significant differences vs. Group 4

**Table 2:** Follicular development, fertilization and embryo quality in various groups of in vitro fertilization cycles with different serum estradiol levels

Outcome	Group 1 (n=243)	Group 2 (n=393)	Group 3 (n=231)	Group 4 (n=256)
Number of follicles >15mm	4.4 ± 1.9#	6.9 ± 2.6#	9.3 ± 3.2#	12.4 ± 3.7
Number of total oocytes retrieved	5.3 ± 3.0#	8.3 ± 3.6#	10.9 ± 4.5#	15.3 ± 6.1
Number of mature oocytes retrieved	4.3 ± 2.6#	6.6 ± 3.0#	8.7 ± 3.8#	12.2 ± 5.1
Maturation rate per oocyte retrieved	0.81 ± 0.19	0.80 ± 0.18	0.80 ± 0.14	0.80 ± 0.15
Cycles with poor oocyte quality	118(48.6%)	186(47.3%)	110(47.6%)	104(40.6%)
Fertilization rate	0.61 ± 0.32	0.62 ± 0.27	0.59 ± 0.26	0.61 ± 0.25
Number of total viable embryos	1.7 ± 1.5#	2.5 ± 1.7#	2.8 ± 1.9#	3.9 ± 3.1
Viable embryo per fertilized oocyte	0.58 ± 0.36#	0.58 ± 0.30#	0.52 ± 0.29	0.49 ± 0.27
Number of good quality embryos	1.2 ± 1.4#	1.8 ± 1.7#	2.0 ± 1.8#	2.9 ± 2.8
Good quality embryos per fertilized oocyte	0.39 ± 0.36	0.38 ± 0.31	0.35 ± 0.29	0.36 ± 0.28

Values are numbers (percentages) of cycles, or mean ± standard deviation.

n = number of cycles of in vitro fertilization

^#^ (*p*<0.01) indicates significant differences from Group 4

**Table 3:** Implantation, pregnancy, and live birth rates of in vitro fertilization cycles, including miscarriage and multiple gestation rates per pregnancy, and incidence of OHSS according to serum estradiol level.

Outcome	Group 1 (n=243)	Group 2 (n=393)	Group 3 (n=231)	Group 4 (n=256)	EC (n=54)
Implantation rate	0.23 ± 0.35	0.20 ± 0.33*	0.21 ± 0.35	0.28 ± 0.38	--
Pregnancy per initiated cycle	72/243 (29.6%)	120/393 (30.5%)	71/231 (30.7%)	82/256 (32.0%)	--
Pregnancy per OR	72/240 (30.0%)	120/387 (31.0%)	71/229 (31.0%)	82/255 (32.2%)	--
Pregnancy per ET	72/196 (36.7%)	120/357 (33.6%)	71/209 (34.0%)	82/186 (44.1%)	--
Live birth per ET	53/196 (27.0%)	83/357 (23.2%)	57/209 (27.3%)	68/186 (36.6%)	--
Miscarriage per pregnancy	17/72 (23.6%)*	29/120 (24.2%)*	7/71 (9.9%)	10/82 (12.2%)	--
Multiple gestation rate per pregnancy	16/72 (22.2%)	25/120 (20.8%)	19/71 (26.8%)	31/82 (37.8%)	--
OHSS	11/243 (4.5%)#	38/393 (9.7%)#	35/231 (15%)	56/256 (22%)	18/54 (33%)

Values are numbers (percentages) of cycles or mean ± standard deviation.

n = number of cycles of in-vitro fertilization

EC: Elective cryopreservation of all embryos

OR: Oocyte retrieval

ET: Embryo transfer

OHSS: Ovarian hyperstimulation syndrome

* (*p*<0.05) and

^#^ (*p*<0.01) indicate significant differences vs. Group 4

**Table 4:** Follicular development, fertilization and embryo quality parameters as a function of serum estradiol level among favorable prognosis IVF patients.

Outcome	Group 1 (n = 97)	Group 2 (n = 226)	Group 3 (n = 146)	Group 4 (n = 195)	EC (n=46)
Number of follicles > 15mm	5.0 ± 2.3#	7.2 ± 2.7#	9.4 ± 3.1#	12.4 ± 3.7	15.3 ± 4.2
Number of total oocytes retrieved	6.0 ± 3.3#	8.4 ± 3.7#	11.0 ± 4.6#	15.3 ± 6.0	21.6 ± 6.1
Number of mature oocytes retrieved	5.0 ± 2.9#	6.6 ± 3.0#	8.9 ± 3.9#	12.3 ± 5.0	17.1 ± 5.4
Maturation per oocyte retrieved	0.82 ± 0.17	0.79 ± 0.18	0.81 ± 0.14	0.81 ± 0.14	0.79±0.11
Cycles with poor oocyte quality	46 (47.4%)	96 (42.5%)	64 (43.8%)	79 (40.5%)	15(32.6%)
Fertilization rate	0.65 ± 0.29	0.61 ± 0.28	0.59 ± 0.27	0.61 ± 0.25	0.61±0.22
Number of total viable embryos	2.1 ± 1.8#	2.4 ± 1.7#	2.8 ± 2.0#	3.9 ± 3.1	6.4 ± 4.3
Viable embryo per fertilized oocyte	0.56 ± 0.33	0.57±0.30*	0.52 ± 0.29	0.48 ± 0.26	0.59±0.29
Number of good quality embryos	1.7 ± 1.7#	1.8 ± 1.7#	2.1 ± 1.9#	3.0 ± 2.9	4.5 ± 4.0
Good quality embryos per fertilized oocyte	0.44 ± 0.35	0.39 ± 0.32	0.37 ± 0.30	0.36 ± 0.28	0.40±0.31

Values are numbers (percentages) of cycles, or mean ± standard deviation.

n = number of cycles of in-vitro fertilization

EC: Elective cryopreservation of all embryos

^#^ (*p*<0.01) indicates significant difference vs. Group 4

**Table 5:** Implantation rate, pregnancy and live birth rates per cycle (including miscarriage and multiple gestation rates) observed in favorable prognosis IVF patients as a function of serum estradiol level.

Outcome	Group 1 (n = 97)	Group 2 (n = 226)	Group 3 (n = 146)	Group 4 (n = 195)
Implantation rate	0.30 ± 0.38	0.24 ± 0.36	0.26 ± 0.37	0.34 ± 0.39
Pregnancy per initiated cycle	39/97 (40.2%)	80/226 (35.4%)	50/146 (34.2%)	69/195 (35.4%)
Pregnancy per OR cycle	39/97 (40.2%)	80/225 (35.6%)	50/145 (34.5%)	69/195 (35.4%)
Pregnancy per ET cycle	39/81 (48.1%)	80/203 (39.4%)	50/130 (38.5%)	69/137 (50.4%)
Live birth per ET cycle	27/81 (33.3%)	57/203 (28.1%)	40/130 (30.8%)	58/137 (42.3%)
Miscarriage per pregnancy	10/39 (25.6%)*	18/80 (22.5%)	4/50 (8%)	8/69 (11.6%)
Multiple gestation per pregnancy	8/39 (20.5%)	18/80 (22.5%)	13/50 (26.0%)	20/69 (29.0%)
OHSS	7/97 (7.4%)*	26/226 (11.5%)*	23/146 (15.9%)	43/195 (21.9%)

Values are numbers (percentages) of cycles, or mean ± standard deviation.

n = number of cycles of in-vitro fertilization

OR: Oocyte retrieval

ET: Embryo transfer

OHSS: Ovarian hyperstimulation syndrome

* (*p*<0.05) indicates significant difference vs. Group 4
